# Development and validation of the HPV-WAK questionnaire for assessing women’s awareness and knowledge in Iran

**DOI:** 10.1371/journal.pone.0340705

**Published:** 2026-01-12

**Authors:** Parisa Ebrahimi, Fatemeh Pourmotahari, Hamed Rahimi

**Affiliations:** 1 Student Research Committee, Dezful University of Medical Sciences, Dezful, Iran; 2 Department of Public Health and Community Medicine, School of Medicine, Dezful University of Medical Sciences, Dezful, Iran; 3 Infectious and Tropical Disease Research Center, Dezful University of Medical Sciences, Dezful, Iran; Tarbiat Modares University Faculty of Medical Sciences, IRAN, ISLAMIC REPUBLIC OF

## Abstract

**Background:**

Evidence indicates that knowledge about Human Papillomavirus remains limited in Iran. This study aimed to develop and psychometrically validate the Human Papillomavirus-related women’s awareness and knowledge (HPV-WAK) questionnaire for Iranian women.

**Methods:**

A mixed-methods study was conducted. First, an item pool was created through a rapid scoping review. Subsequently, the questionnaire’s psychometric properties were assessed, including face validity, content validity (CVR/CVI), and hypothesis testing for construct validity (known groups comparison). Reliability was evaluated via internal consistency (KR-20) and inter-rater reliability (kappa). A cut-off score was determined using ROC analysis.

**Results:**

From an initial 551 items, 122 were selected for the item pool. Following translation and cultural adaptation, a 50-item version underwent face validity assessment, leading to revisions of 5 items. The final 29-item questionnaire showed strong content validity (S-CVI/Ave = 0.97; S-CVI/UA = 0.83) after eliminating 21 items with low CVR (<0.62). construct validity was confirmed via known groups comparison (P < 0.001). The questionnaire demonstrated moderate internal consistency (KR-20 = 0.757) and excellent inter-rater reliability (Kappa = 0.854). The optimal cut-off score was 26.

**Conclusions:**

The HPV-WAK is a valid and reliable questionnaire with strong psychometric properties, making it suitable for use by public health professionals to assess HPV knowledge, identify gaps, and evaluate interventions among Iranian women of childbearing age.

## Background

Sexually transmitted diseases (STDs) have an impact on sexual health [1], which is an important part of general health, well-being, and quality of life [2,3]. STDs and their complications contribute significantly to costs, disability, and death worldwide [4]. The World Health Organization (WHO) estimates that nearly 1000000 individuals 15–49 years old obtain a Sexually Transmitted Infection (STI) every day [5]. From 1990 to 2021, the global incidence of STDs rose by approximately 58.38%, reaching about 289 million cases [6]. Therefore, STDs represent a worldwide health priority because of their considerable effects on communities [5]. Consequently, efforts to eliminate STDs by 2030 are considered one of the new strategies of the WHO Global Health Sector [7].

Among sexually transmitted diseases, HPV is the most prevalent [8]. This virus is transmitted mainly through unprotected intercourse [9], and approximately all sexually active people will encounter in their lives [10]. It is estimated that approximately 80% of women who engage in sexual activity are in contact with the HPV at least once during their lifetime [9]. The virus causes a significant disease burden, including cancer and warts [11–13]. Most importantly, HPV is the main cause of cervical cancer [5,14]. HPV and its related diseases lead not only to physical consequences but also to numerous psychological and social issues for patients, families, and society [15]. The negative impacts on sexual health, reproductive health, and emotional health, along with decreased sexual desire, marital problems, inadequate satisfaction with marital life, the propensity for risky behaviors, personal and psychological issues, depression, the emergence of negative emotions, and other psychiatric disorders, are among the consequences of this virus [16–18].

However, evidence indicates that knowledge about HPV remains limited [19]. This is particularly true in Islamic countries, where information about sexual health is not easily accessible. In these countries, barriers exist at various levels, ranging from individual to cultural and religious [20]. For example, a study conducted in Oman showed that not only parents but also health care providers have poor knowledge about HPV. Less than 25% of participants had heard of HPV [21]. Another study in Saudi Arabia also showed that there is a significant gap in knowledge and awareness related to HPV and its vaccine. A lack of knowledge was identified as the primary barrier preventing participants from receiving the HPV vaccine [22]. The study by Rafiei et al., like previous studies conducted in Iran, assessed the level of women’s knowledge and awareness about HPV as poor [23]. In Iran, cultural constraints and religious beliefs hinder the development of a coherent public educational program on HPV [23]. Consequently, HPV poses a significant threat to women’s health as an STD [24]. Hence, appropriate policies, plans, and measures must be implemented to increase Sociocultural sensitization and improve preventive behaviors [25]. In this regard, as a first step, it is essential to assess the degree of awareness and knowledge of the disease via a reliable instrument [26,27].

There are many tools available for this purpose, but they are not appropriate for non-Western societies, especially those that are Islamic in orientation. The direct application of Western-developed questionnaires in Islamic societies is methodologically problematic due to profound cultural, religious, and socio-legal divergences, which can compromise conceptual, semantic, and functional equivalence [28,29]. In this regard, there is a major methodological gap in the literature on HPV knowledge assessment in Iran. Although translated instruments have been used in some studies [30–32], their psychometric reporting is frequently restricted to internal consistency and basic face and content validity. Importantly, for the Persian versions of the instruments, these studies consistently show no signs of rigorous cultural adaptation or strong construct validity analysis. The need for the current study is highlighted by the lack of a thoroughly validated and culturally grounded instrument for the Iranian population. Furthermore, discussions about sexual behavior are frequently subject to sociocultural restrictions in religiously oriented societies like Iran. As multiple studies indicate, direct questions about sexual activity may impede responses for several reasons, including the potential for response bias, a decreased willingness to engage honestly out of fear of shame and stigma, and a conflict with social and religious norms [33–35].

As a result, careful cultural adaptation is needed in the design of assessment tools in this field, including vocabulary revision and the use of indirect phrases. Therefore, this study tried to develop a valid and reliable tool for measuring Iranian women’s knowledge and awareness of HPV within an Islamic social context, given the pressing need to develop valid tools for measuring awareness as a first and crucial step for any Theory of Planned Behavior-based interventions in the health sector.

## Materials and methods

### Aim and design

This study aimed to design and validate an HPV-related women’s awareness and knowledge (HPV-WAK) questionnaire. The study was carried out with the approval of the Ethics Committee of the Dezful University of Medical Sciences (IR.DUMS.REC.1402.071). It employs both quantitative and qualitative methods to generate items, design questionnaires, reduce items, and conduct psychometrics. It is based on relevant guidelines, including the PRISMA-ScR guidelines [36] for rapid scoping reviews and the COSMIN guidelines [37] for quantitative methods. All procedures were performed following these guidelines and relevant regulations. The study was conducted from March 10, 2024, to October 21, 2024. The research process followed this timeline: literature review and item generation (March-April 2024), translation and cultural adaptation (May 2024), and questionnaire psychometric testing, including participant recruitment and data analysis (June – October 2024). The study involved the participation of appropriate specialists and women of childbearing age (15–49 years) in Iran.

### Sample

The research sample was targeted based on entry criteria. In the face validity section, 17 individuals from the target group (women aged 15–49) with the lowest literacy levels (Diploma and less than a diploma), who had visited the comprehensive health services center, were selected to assess the comprehensibility of the questions. Saturation was reached with the participation of 17 individuals. Ten professionals participated in the content validity section. The criteria for entering the study included relevant education (Such as a Ph.D. degree in Infectious disease, gynecology, nursing, and health education and promotion), at least 5 years of related work experience, a history of conducting related research, and willingness to participate. All participants were working in a comprehensive health services center, hospital, behavioral diseases center, and faculty members of medical and nursing schools at the time of data collection. Reluctance to cooperate was also considered a reason for withdrawal from the study. Written informed consent was obtained from all participants. For minors, the consent of parents or guardians was considered.

The known-groups approach was applied to assess the questionnaire’s construct validity by comparing two distinct groups. The informed group (58 individuals) was chosen through purposive sampling from four specialized categories: faculty members of infectious disease, gynecology medicine, and health education at Dezful University of Medical Sciences, medical practitioners employed in Dezful health-care facilities and hospital, nursing personnel in gynecological and infectious diseases units of Dezful Teaching Hospital, and public health and health education specialists operating in Dezful comprehensive health services centers. The uninformed group (62 individuals) was selected from the referrals to Dezful comprehensive urban health services centers utilizing multistage random cluster sampling, ensuring that none of the individuals in this group possessed a background in medical sciences or had participated in HPV educational initiatives. The inclusion criteria for both groups encompassed ages 25–55 years, written informed consent, and the absence of sexually transmitted infections. To enhance diagnostic validity, purposive sampling of the informed group was executed based on explicit parameters, including a minimum of 5 years of specialized professional experience in the domain of HPV and possession of pertinent articles or training programs. A power analysis was conducted using the ***pwr*** package in R software. Based on the large effect size (Cohen’s d = 1.97) observed in the data and an alpha level of 0.05, the analysis confirmed that the sample size of 120 participants would provide statistical power exceeding 0.90. The appropriate sample size for reliability testing (n = 36) was determined based on the null hypothesis, an alpha coefficient of 0.5, and the total number of questionnaire items [38].

### Implementation

#### Step 1: Questionnaire development.

**Literature review and item generation:** The first stage of the study involves secondary studies, which utilize a rapid scoping review approach to gather evidence. Initially, studies related to knowledge and awareness of HPV were searched in the PubMed and Embase databases without a time limit in 2023. A combination of relevant keywords based on MESH was employed for the search. The search strategies are outlined in the S1 Table. The studies obtained through two stages were first assessed by reviewing the titles and abstracts, followed by an examination of the full texts by two individuals independently. When there was conflict, a consensus was reached for the final decision. When further resolution was needed, the input of a third person was sought. As is common in many scoping review studies, this study did not assess the quality of the articles [39]. Finally, data were extracted according to the research objectives. The content analysis method and contractual content analysis approach were employed for data analysis, allowing for inductive classification. Initially, the extracted items (after removing duplicates) were encoded as semantic units and constituted the pool of items. The codes were subsequently read multiple times and categorized based on semantic similarity.

**Translation and cultural adaptation:** The items were translated using the forward-backward translation method. Initially, two native Persian speakers independently translated the items into Persian. One of the translators was knowledgeable about the topic and relevant conceptual frameworks, which helped identify discrepancies. After comparing their translations to find differences, the translators and the research team collaboratively prepared the final Persian version. Then, two additional translators independently back-translated the Persian version into English. Ultimately, a multidisciplinary expert panel was formed to assess the items and develop the questionnaire. The panel consisted of seven members, including an infectious disease specialist, a gynecologist, a general practitioner, a public health expert, a nurse experienced in designing questionnaires, a research team member, and one of the translators. The selection criteria included: a minimum of 5 years of professional experience in their field, and specific expertise related to HPV, questionnaire development, and cross-cultural adaptation. This panel carefully reviewed both the original and translated items, resolving disagreements to reach consensus. In the item generation process, the cultural adaptation approach was prioritized over direct translation due to the conceptual and cultural diversity of the target population and the heterogeneity of the foundational studies. This was undertaken to preserve conceptual relevance, ensure accurate comprehension, and establish the ecological validity of the instrument within the specific socio-cultural context of the study population.

The panel then evaluated items from the pool to develop the first draft of the questionnaire, considering criteria such as alignment with the study’s goals, cultural relevance for the target population, comprehensibility, and the items that frequently appeared in previous research and other instruments. As needed, panel members were allowed to add, remove, combine, or modify items as needed. They also discussed and decided on the questionnaire’s format, the format for individual items, and the overall length. The first draft of the HPV-WAK questionnaire was finalized after the panel agreed on the items to include.

#### Step 2: Questionnaire Psychometry.

In this step, the validity (face, content, and construct) and reliability of the HPV-WAK questionnaire were investigated both quantitatively and qualitatively.

**Face validity:** The face validity of the questionnaire was checked both quantitatively and qualitatively, involving the participation of 17 individuals with the lowest literacy levels. In the qualitative method, cognitive interviews were conducted to ensure the items’ difficulty and ambiguity. To this end, challenging, ambiguous, and disproportionate aspects were identified from the participants’ perspectives. The research team then reread and modified the items multiple times. The quantitative approach also examined the comprehensibility of the items via a five-point Likert scale (completely understandable, understandable, moderately understandable, not understandable, and completely incomprehensible). The items’ significance was evaluated via the item impact method (Formula 1), and those with an impact score under 1.5 were revised.


Item Impact Score =Frequency (%)  Importance (1)

**Content validity:** A combination of quantitative and qualitative approaches was employed to determine content validity. For the qualitative method of reviewing content validity, 10 experts were asked for opinions and suggestions on the correct writing style, grammar compliance, logical wording, word choice, item placement, and scoring. For quantitative analysis, two metrics, the content validity ratio (CVR) and the content validity index (CVI), were subsequently applied. The experts were therefore asked to score items on a three-degree spectrum (necessary, useful but not necessary, and not necessary) to determine the CVR. On the Basis of Formula 2, the CVR ranges from 1 to −1; a higher value indicates that experts agree that the item is necessary. In this formula, N is the overall number of specialists, and Ne is the number of people who have chosen the necessary option. The numerical value of the CVR is determined by the Lawshe table, according to which items with a CVR greater than or equal to 0.62 (based on the evaluation of 10 experts) are selected [40].


$$CVR=(Ne-\frac{N}{2})/(\frac{N}{2})$$ (2)

The CVI was subsequently determined via Waltz and Basil’s content validity index. For this purpose, participants used a four-point scale based on the “relevance” criterion. Each item’s CVI score was assessed by dividing the number of participants who gave scores of three and four by all participants (Formula 3). The Lynn table states that the requirement for approving an item with the participation of 10 evaluators is at least 0.8, and values below that should be excluded [41].


$$\;I-CVI=\frac{\text{Number}\;\text{of}\;\text{participants}\;\text{giving}\;\text{a}\;\text{score}\;\text{of}\;\text{three}\;\text{or}\;\text{four}}{\text{Overall}\;\text{number}\;\text{of}\;\text{participants}}$$ (3)

Next, the probability of the random correlation coefficient (Pc) was initially computed via Formula (4) and the approach of Polit et al., where N represents the number of specialists and A denotes the number of consensuses. The modified kappa coefficient (k*) was then calculated via Formula (5) for each item. The evaluation of k* values was based on the standards of Cicchetti and Sparrow (1981) and Fleiss (1981), which categorize values as less than 0.4 (weak), 0.4 to 0.59 (fair), 0.6 to 0.74 (good), and more than 0.74 (excellent) [42,43]. In this study, items with k* values greater than 0.74 were considered acceptable.


$$p_{c}=\left[\frac{N!}{A!(N-A)!}\right]\times 0.5^{N}$$ (4)

$$k^{*}=\frac{\text{I}-\text{CVI}-p_{c}}{\text{1}-p_{c}}$$ (5)

The instrument’s content validity was calculated via the Scale-Level Content Validity Index/Average (S-CVI/Ave) and Scale-level Content Validity Index/Universal Agreement (S-CVI/UA) indices. Consequently, the S-CVI/Ave was derived from the I-CVIs’ mean of all instrument items. The S-CVI/UA was obtained by taking the proportion of items rated three or four by all specialists (Formula 6). For an instrument to demonstrate excellent content validity, it is recommended that the S-CVI/Ave be at least 0.9 and that the S-CVI/UA be at least 0.8 [42].


$$S-CVI/UA=\frac{\text{Items}\;\text{that}\;\text{obtained}\;\text{a}\;\text{rating}\;\text{of}\;\text{three}\;\text{or}\;\text{four}\;\text{by}\;\text{all}\;\text{participants}}{\text{Overall}\;\text{number}\;\text{of}\;\text{items}}$$ (6)

**Construct validity (known group):** The known-groups method was used to determine the construct validity of this instrument. This method examines the instrument’s ability to gauge differences between distinct groups (extremist groups) that are expected to vary in a particular feature [44]. The study hypothesized that individuals with medical education and those working in the health system possess greater knowledge and awareness about HPV than do individuals without these characteristics. Thus, the questionnaire was planned to be distributed via convenience sampling to 120 women (60 in the informed group and 60 in the uninformed group) to assess known-groups validity.

**Reliability:** Given the dichotomous (Yes/No) nature of the items, the Kuder-Richardson Formula 20 (KR-20) was used to calculate internal consistency, as it is specifically designed for this type of data [45]. The KR-20 reliability coefficient is considered poor if it is less than 0.50, moderately reliable if it is between 0.50 and 0.80, and highly reliable if it exceeds 0.80 [46]. Additionally, the kappa index was employed to evaluate inter-rater agreement. Three raters independently assessed the instrument in this study, and the average inter-rater kappa coefficient was calculated. In terms of clinical significance, a kappa coefficient of less than 0.4 is considered poor, a value between 0.4 and 0.59 is fair, a value between 0.6 and 0.74 is good, and a value between 0.75 and 1 is excellent [47,48].

**Fig 1:**
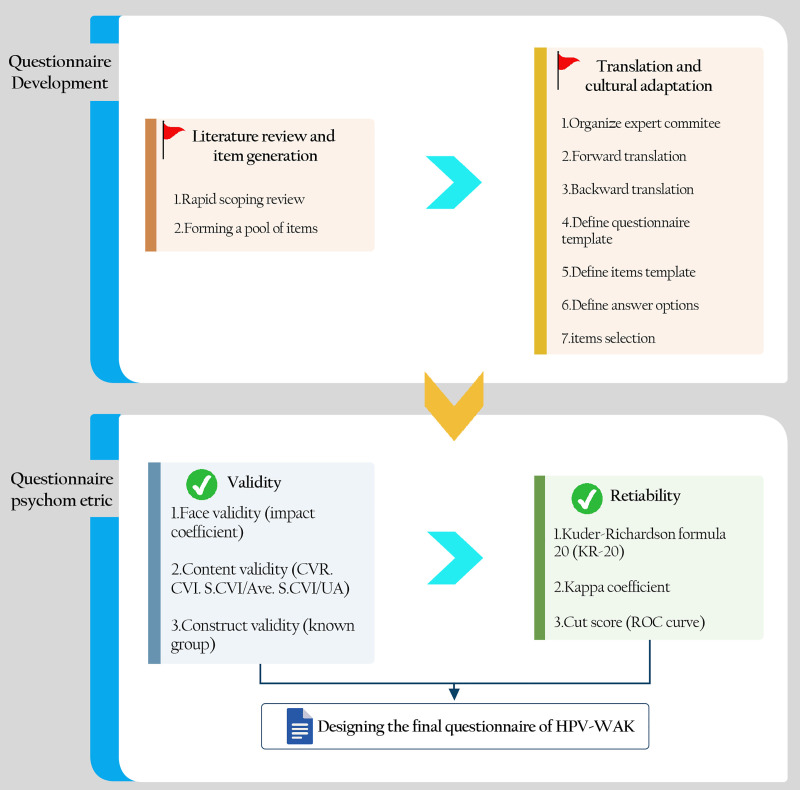
Questionnaire development and psychometric processes.

**Cut score:** The appropriate cut-off score for the HPV-WAK questionnaire in this population was identified via receiver operating characteristic (ROC) curve analysis. The sensitivity, positive predictive value, specificity, negative predictive value, and area under the receiver operating characteristic curve (AUC) with a corresponding 95% confidence interval were computed to evaluate the questionnaire’s diagnostic accuracy. The development of the questionnaire and psychometric processes is illustrated in Fig 1.

### Data analysis

In the initial phase, the contractual content analysis method was utilized to synthesize extracted items from the literature. In the second step, descriptive indicators such as rates and percentages were employed for the demographic data. The validity and reliability indicators were evaluated to measure the psychometrics of the instrument. The facial validity (impact coefficient), content validity (CVR, I-CVI, S-CVI/Ave, and S-CVI/UA), and construct validity (known groups) were calculated for instrument validity as described. Owing to the lack of data normality (Kolmogorov-Smirnov test), the Mann-Whitney test was conducted at a significance level of 0.05 to analyze the data of known groups. The KR-20 and the kappa index were also used to measure the instrument’s reliability. Data analysis was conducted via Microsoft Excel, SPSS V.26, and R V.4.3.3. No missing data were encountered in this study, as all distributed questionnaires were returned fully completed. Consequently, no data imputation or deletion techniques were required.

## Results

### Stage 1

At this stage, 56 studies were selected and 551 items were extracted. The characteristics of these studies that contributed to the item generation process are detailed in the S1 Table. After removing duplicate items, 253 items remained. Items that overlapped in content and purpose but differed only in wording were preserved. By evaluating the remaining items, we excluded 74 that were not aligned with the study objectives, fell outside the field of knowledge and awareness, or targeted a different group, resulting in 179 items remaining. After screening the overlapping items, 122 unique items remained and were constructed into a pool of items. Finally, the initial pool of 122 items was reduced to 50 items by the expert panel to eliminate conceptual redundancy, retaining the single most clear and comprehensive item from clusters measuring identical variables.

The expert panel ultimately selected 50 items for the initial draft of the HPV-WAK questionnaire after translating the items using the forward and backward translation process. All aspects of the disease, including prevalence, risk factors, transmission, symptoms, prevention, screening and diagnosis, consequences, and treatment, are addressed by the questionnaire’s items. The expert panel agreed that the questionnaire should be self-administered, contain closed-ended items, and have “true,” “false,” and “don’t know” as response options.

### Stage 2

**Face validity:** In the qualitative section, 5 items were revised in the wording. In the quantitative section, all the items were validated, as every one of the 50 items had an impact factor greater than 1.5. The participants included 17 women, of whom 88% held a diploma, 76% were married, 59% were city residents, and their average age was 37.5 (±7.3) years, ranging from 21 to 43 years.

**Content validity:** In the qualitative section, all the items received approval. However, according to the CVR analysis, 21 items were removed because their CVR scores were below 0.62 (S2 Table). Therefore, the instrument consisted of 29 items, with CVR values ranging from 0.8 to 1.0 (Table 1). The CVI analysis revealed that the scores for all the items were greater than 0.8, allowing their retention. Additionally, the S-CVI/Ave and S-CVI/UA values were computed at 0.97 and 0.83, respectively, indicating excellent content validity for the entire instrument (Table 1). At this stage, 10 specialists participated, including 7 Ph.D. holders in health education and promotion, 2 Ph.D. holders in nursing, and a subspecialist in infectious diseases. The average age of the participants was 43.7 years (±4.2), with 60% being female and 90% married.

**Table 1:** Content validity results of retained items of the HPV-WAK questionnaire.

	Item	CVR	I-CVI	P_c_	K^*^	Evaluation
1	Human papillomavirus infection is very rare.	0.8	0.8	0.044	0.79	Retained
2	Men are not at risk of getting HPV.	0.8	1	0.001	1	Retained
3	Early initiation of sexual activity reduces the risk of getting HPV.	0.8	1	0.001	1	Retained
4	The risk of obtaining HPV is increased by having several sexual partners.	1	1	0.001	1	Retained
5	Being in a monogamous relationship eliminates the risk of getting HPV.	1	1	0.001	1	Retained
6	Sexual interaction can transmit HPV.	1	1	0.001	1	Retained
7	The risk of getting HPV is reduced by using a condom.	0.8	1	0.001	1	Retained
8	HPV can only be transmitted from a carrier who has obvious symptoms.	1	1	0.001	1	Retained
9	During pregnancy and childbirth, HPV can be passed from mother to child.	0.8	1	0.001	1	Retained
10	Skin contact in the genital area can spread HPV.	0.8	1	0.001	1	Retained
11	The majority of HPV-positive individuals are unaware of their condition because it does not show obvious signs or symptoms.	1	1	0.001	1	Retained
12	Abnormal bleeding between periods is a symptom of cervical cancer.	0.8	0.9	0.01	0.9	Retained
13	Only sexually active women should get the HPV vaccine.	1	1	0.001	1	Retained
14	Every sexually transmitted infection can be prevented with the HPV vaccine.	0.8	1	0.001	1	Retained
15	One way to prevent cervical cancer is to get the HPV vaccine.	1	1	0.001	1	Retained
16	If a person has been vaccinated, they will never get HPV.	0.8	0.9	0.01	0.9	Retained
17	A person who has been infected with HPV should still get vaccinated.	0.8	1	0.001	1	Retained
18	Vaccination prevents certain types of HPV.	0.8	1	0.001	1	Retained
19	Good sexual hygiene alone can prevent cervical cancer.	0.8	1	0.001	1	Retained
20	Women who have received the HPV vaccine do not need to have routine screening exams.	0.8	1	0.001	1	Retained
21	The HPV test and Pap smear can be conducted together.	1	1	0.001	1	Retained
22	A woman will undoubtedly get cervical cancer if her HPV test is positive.	0.8	0.9	0.01	0.9	Retained
23	Cervical cancer is curable if caught early.	0.8	1	0.001	1	Retained
24	The only way to diagnose cervical cancer is with a Pap smear.	0.8	1	0.01	1	Retained
25	HPV can cause genital warts.	1	1	0.001	1	Retained
26	HPV only causes cervical cancer in women.	1	1	0.001	1	Retained
27	HPV always causes cancer.	0.8	0.9	0.01	0.9	Retained
28	There is no specific treatment for HPV.	0.8	1	0.001	1	Retained
29	The only cure for cervical cancer is to remove the uterus.	1	1	0.001	1	Retained
**Total**		S-CVI/Ave				**0.97**
		S-CVI/UA				**0.83**

**Construct validity:** In the informed group, 66% were in medicine and 28% were in nursing, with an average age of 27.86 (±3.1); 60% were married, and all were employed in the hospital. In the uninformed group, 78% had a diploma, with an average age of 29.32 years (±4.5); 82% were married, and 65% were housewives. The known groups (informed/uninformed) differed significantly based on the findings of the Mann-Whitney test, which was used to compare them (P = 0.000). The effect size was calculated as r = 0.801, indicating a large effect (Table 2).

**Table 2:** The construct validity of the HPV-WAK questionnaire.

Variable	Number	Mean (±SD)	Median	Effect size	P-value
Informed Group	58	26.79 (±0.97)	27	0.801	0.000
Uninformed Group	62	20.92 (±4.09)	22		

**Reliability:** With a KR-20 reliability of 0.757, the instrument demonstrated acceptable reliability. A very high level of agreement among the raters in assessing the instrument was evidenced by the average kappa coefficient among the raters, which was 0.854. Internal consistency (KR-20) was calculated using a subset of 36 participants from the main validation sample, as determined by our a priori reliability sample size calculation.

**Fig 2:**
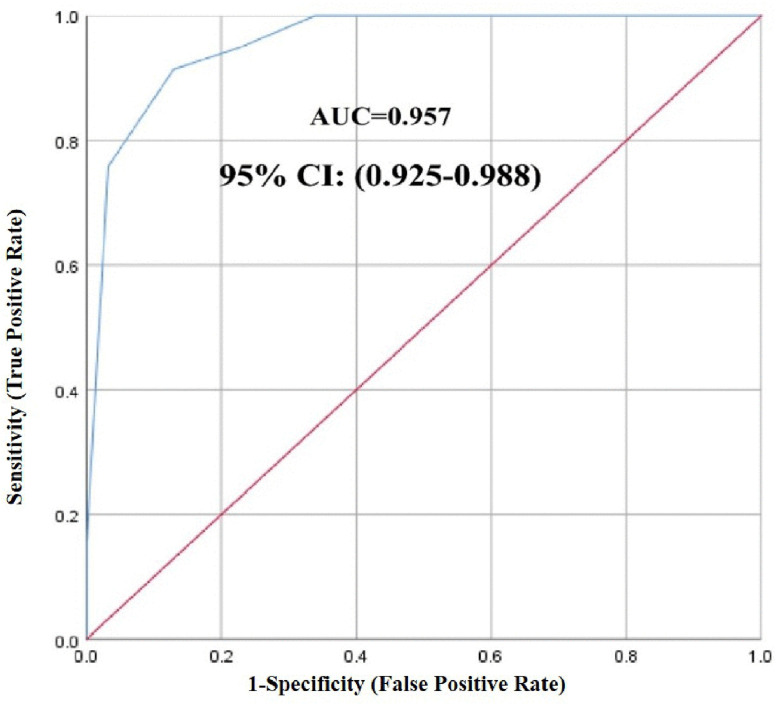
ROC curve for the HPV-WAK questionnaire.

**Cut score:** According to the ROC analysis, the AUC of the HPV-WAK questionnaire was 0.957 (Fig 2). Using Youden’s index, we identified 25.5 as the optimal cut-off score, balancing sensitivity (91.37%) and specificity (87.09%). This threshold showed adequate predictive performance, with positive and negative predictive values of 86.88% and 91.25% respectively (Table 3). Since the questionnaire’s total score is discrete (allowing only whole-number values), the optimal threshold of 25.5 was pragmatically rounded to 26. This adjustment maintained both statistical validity (AUC = 0.892, 95% CI: 0.837–0.948) and practical applicability. Thus, scores ≥ 26 indicate sufficient HPV knowledge and awareness. The comprehensive process of questionnaire development and validation is summarized in Table 4.

**Table 3:** The diagnostic accuracy of the cut-off score of the HPV-WAK questionnaire.

HPV-WAK questionnaire	Cut score	Sensitivity %	Specificity %	PPV %	NPV %	AUC (CI 95%)
25.5	91.37	87.09	86.88	91.52	0.957 (0.925-0.988)
26	91.13	87.09	90.10	88.40	0.892 (0.837-0.948)

PPV: Positive Predictive Value, NPV: Negative Predictive Value, AUC: Area under the receiver operating characteristic curve, CI: Confidence Interval

**Table 4:** Summary of questionnaire development and item reduction process.

Phase	Objective	Method	Outcome
**Literature review and Item generation**	To create a comprehensive initial item pool	Rapid scoping review	122 initial item generation
**Initial item reduction**	To cultural adaptation and eliminate conceptual redundancy	Expert panel	50 items retained for initial questionnaire
**Face validity assessment**	To ensure items’ difficulty and ambiguity, and comprehensibility from the target population’s perspective	Quantitative method: five-point Likert scale Qualitative method: cognitive interviews	50 items had an impact factor greater than 1.5. The wording and phrasing of the items were finalized based on feedback.
**Content validity assessment**	To ensure necessary relevance, clarity, and cultural appropriateness	Quantitative (CVR, CVI, S-CVI/Ave, and S-CVI/UA) and qualitative evaluation by a multidisciplinary expert panel (n = 10).	21 items were removed and 29 items retained.
**Construct validity assessment**	To test the questionnaire’s ability to discriminate between known groups	Known-groups: independent samples t-test comparing scores between informed/uninformed groups	Significant difference in scores between groups (p < o.01) confirmed the questionnaire’s construct validity
**Reliability**	to evaluate the internal consistency of the final questionnaire	The Kuder-Richardson Formula 20 (KR-20) was used	The KR-20 reliability rating of 0.757 is considered acceptable.
**Cut-off score**	To establish an optimal score for screening/classification	Receiver Operating Characteristic (ROC)	A cut-off score of 26 was identified, yielding a sensitivity of 91.13% and specificity of 87.09% (AUC = 0.892)

## Discussion

Determining individuals’ awareness and knowledge plays a key role in developing the right strategy for effective health planning. Many sexually transmitted diseases arise from insufficient awareness and misconceptions. Studies have shown that awareness of human papillomaviruses positively impacts attitudes toward vaccination, vaccine acceptance, and the number of doses received [23,49,50]. Therefore, considering the function of precancerous HPV and the value of avoiding it, measuring and enhancing society’s awareness and knowledge in this area is essential, especially for women [32]. On the other hand, the questionnaire, which is one of the most extensive methods for gathering data from respondents, is necessary to ensure accurate development and establish its validity and reliability. Specifically, such a process guarantees the successful implementation of preventive measures against communicable diseases in interventional studies [51]. Thus, this study aimed to develop and validate a new instrument to measure women’s awareness and knowledge of HPV and its vaccine in Iran. The HPV-WAK questionnaire was designed with 29 items in Persian. The face and content validity of the questionnaire were confirmed through quantitative and qualitative methods, whereas the construct validity was assessed via known groups and evaluated for internal consistency and inter-rater agreement.

Initially, on the basis of findings from the rapid scoping review, 122 items were selected as a pool of items. The initial questionnaire was ultimately designed with 50 items, following the research team’s examination of overlapping themes and cultural fit. For qualitative facial validity, only 5 items were edited for clarity, and the impact coefficient of all the items exceeded the cut-off point. Therefore, the facial validity of the designed instrument is confirmed. Du et al. [52], Khandpur et al. [53] Abdullah et al. [54], and Sukrong et al. [55] have used face validity in their studies to examine the validity of the awareness and knowledge questionnaire. The CVR and CVI indicators were computed in the quantitative content validity stage. Initially, 21 items were removed on the basis of the CVR calculations, and then the remaining items (29 items) were evaluated for CVI. The findings demonstrated that the I-CVI of all 29 items was equal to 0.8 or higher, confirming their validity. Additionally, the S-CVI/Ave and S-CVI/UA were calculated for the entire questionnaire and were found to be acceptable (0.97 and 0.83, respectively). According to Polit et al., who recommend values greater than 0.9 and 8.8 for the S-CVI/Ave and S-CVI/UA [42], respectively, this instrument demonstrates high content validity.

Construct validity was assessed via the known groups’ method, which revealed a significant difference between known groups. Therefore, individuals with medical education and those working in the health system exhibited greater awareness and knowledge than those without these qualifications did. Azraii et al. similarly applied the known groups method to assess the construct validity of the knowledge, awareness, and function questionnaire for family hypercholesterolemia (FH KAP). Their findings indicated that the mean awareness and knowledge score of primary care physicians with a postgraduate degree was significantly higher than that of primary care physicians without a postgraduate degree [56]. Baş and Ursavaş utilized the known groups method to develop a breast cancer awareness measurement questionnaire in Turkish. Their results revealed that health professionals’ awareness significantly surpassed that of women in the general population regarding symptoms and the frequency of breast examinations. However, the groups did not significantly differ concerning correct answers to the query “risk of age-related breast cancer” [57]. While factor analysis is commonly used to examine the construct validity of questionnaires with numerical or continuous responses, such as the Likert scale, it is not recommended for questionnaires with dichotomous scoring [56]. Therefore, the decision to choose the known-groups method for construct validity in the present research was due to the binary nature of the responses.

Internal consistency was assessed by applying the KR-20 reliability coefficient because the HPV-WAK questionnaire is dichotomous [58]. The internal correlation coefficient (ICC) of the KR-20 showed moderate reliability (0.757) for the questionnaire. In a similar study, Azraii et al. also used the KR-20 reliability coefficient method to evaluate the reliability of the FH KAP tool, and the instrument’s reliability was assessed at a moderate level [56]. Kurniawan et al. also used the KR-20 method to measure the reliability of the Indonesian version of the HIV-KQ-18 questionnaire, which assesses general knowledge about HIV/AIDS [59]. In contrast, Waller et al. assessed the internal consistency of the human papillomavirus knowledge questionnaire (HPV-KQ) using reliability analysis, resulting in very good reliability (α = 0.82). They additionally applied item response theory (IRT) to analyze individual items, aiming to improve the questionnaire; discrimination (0.5 to 2.10) and difficulty (1.5 to 3.5) levels were deemed satisfactory [60]. Moreover, after cultural modifications (removing three sensitive questions and changing sexual behavior-related phrases, Demir’s validation study of the HPV-KQ questionnaire in Turkish showed that the instrument had strong psychometric properties, including excellent content validity (CVI = 0.92) and acceptable construct validity (CFI = 0.91). it also had a Cronbach’s alpha of 0.81 and a test-retest reliability of 0.89, further confirming its reliability [61]. These findings support the current study and highlight how importance of adapting HPV questionnaires for cultural differences. By maintaining the core structure of basic knowledge, the current questionnaire (HPV-WAK) incorporates specific cultural adjustments for Iranian society, as evidenced by the results. The HPV-WAK demonstrated acceptable reliability, with a KR-20 value of 0.757. Nonetheless, future studies may further improve internal consistency by refining or removing items with suboptimal performance, as identified through item-total correlations and difficulty index analyses. Moreover, broader cognitive interviews and pilot testing with more diverse populations could enhance item clarity and strengthen the overall reliability.

An excellent level of inter-rater agreement was indicated by the kappa coefficient of 0.854. Thus, this instrument demonstrates appropriate reliability and sustainability over time to assess women’s knowledge and awareness of HPV. Azraii et al. also used Cohen’s kappa in their study to examine the reliability of the test-retest; it was excellent for the dimensions of knowledge and awareness (0.818 and 0.810, respectively) and was well evaluated for the entire questionnaire (0.796) [56].

The HPV-WAK questionnaire’s ability to address knowledge and awareness in the field of sexual health sexual health in Iran, despite cultural and religious barriers, was a key accomplishment. This achievement was made possible by incorporating several distinctive features: (1) alignment with familial and religious values, (2) linguistic adaptation to match the literacy levels of respondents, (3) focus on preventive health aspects rather than sexual behaviors (e.g., emphasizing transmission routes instead of high-risk sexual behavior), and (4) compatibility with Iranian healthcare infrastructure, including implementation by minimally trained health workers and accommodation of counseling limitations within local health centers. Specifically, during the framing of the items, considerable emphasis was placed on the cultural sensitivities inherent in Iranian society, which is Muslim.

In particular, direct questions about uncommon sexual behaviors may elicit non-responses within the context of Iranian culture. For instance, the omission of the statement “HPV is transmitted through oral/anal sex” [62] was a conscious decision and part of cultural adaptation. This decision was based on the understanding that such forms of sexual engagement are profoundly influenced by cultural, legal, religious, and medical considerations, and are regarded as unconventional and taboo within Iranian society [35,63]. Most importantly, the design, which focuses on prevention and transmission and avoids direct questions about sexual behaviors, is specifically designed to reduce social desirability bias. The researchers believe that social desirability bias is a major challenge in sexual health studies, as participants tend to give socially acceptable answers rather than truthful answers [33,35]. By eliminating questions that may cause shame or stigma, in contrast to direct translations of non-native instruments, the questionnaire provides a safer space for honest responses, thereby increasing the validity of the data collected. Importantly, while demonstrating cultural appropriateness, the HPV-WAK questionnaire also maintains scientific rigor by thoroughly addressing all relevant aspects of the disease.

While ROC analysis originated in diagnostic medicine, it has been well-established in psychometric research for determining optimal cut-offs in assessment tools. Our application to identify a knowledge threshold (score = 26) serves as a research-oriented classification method, not a clinical diagnostic. This approach is consistent with established practices in educational and health psychology for group differentiation [64,65]. For example, Naqvi et al. used ROC analysis in designing the rheumatoid arthritis knowledge assessment scale [66].

In general, the HPV-WAK questionnaire consists of 29 questions that provide “correct”, “wrong”, and “I do not know” answers. The scoring of the HPV-WAK questionnaire is as follows: one point for a correct answer, and zero points for “wrong” and “I do not know” answers. Thus, the score range is 0–29, where greater awareness and knowledge of HPV are marked by higher scores, and vice versa. Sixteen questions (1, 2, 3, 5, 8, 13, 14, 16, 19, 20, 22, 24, 26, 27, 28, and 29) were designed negatively (as wrong), so they were scored in reverse (S3 Table). This design, which included both positively and negatively worded items, was chosen in accordance with recognized survey technique practices to avoid acquiescence bias [67,68]. The ROC analysis indicated that the cut-off score of the questionnaire was 25.5. Therefore, a score above 25.5 indicates strong awareness and knowledge about HPV, whereas a score below 25.5 signifies poor awareness.

Several key points can be highlighted regarding the benefits, applications, and scientific implications of this study and the resulting questionnaire:

**Increasing public awareness:** The questionnaire serves as an effective instrument for evaluating and promoting women’s understanding of the HPV virus, the HPV vaccine, and how it connects to cervical cancer. For example, integration into premarital counseling programs, addition to routine assessments at family counseling centers, use in behavioral health and sexual health training workshops, and screening the level of awareness of clients at gynecological and midwifery clinics are suggested.

**Guidance for health programs:** The data from the questionnaire can help health officials design and implement effective training and information programs. The data can be used to develop health strategies, determine and prioritize educational needs, and design targeted health programs. Especially in a country such as Iran, where there is limited information and awareness about HPV due to its cultural and social structure, these data can help sensitize lawmakers, policymakers, and decision-makers and change the paradigm.

**Contributing to future research:** The questionnaire can be utilized as a basic instrument for future studies in various fields related to HPV, including social and cultural impacts, vaccination acceptance, and trends in cervical cancer prevention. It can be employed in cross-sectional studies to gauge women’s awareness and knowledge of HPV and its vaccine. Additionally, it can be utilized in interventional research to evaluate the impact of health education initiatives in improving health literacy levels and tracking changes in HPV awareness.

**Facilitating health policymaking:** The results of this study and the application of a future-designed questionnaire can assist health policymakers in making more informed decisions regarding resource allocation and the development of national HPV prevention programs, ultimately contributing to a reduced cervical cancer burden and improved community health.

**Strengthening international research:** Given that the prevalence of HPV is a global health challenge, presenting this questionnaire can establish a basis for international cooperation in related research. In particular, comparing results across different countries and regions can enhance experience exchange and improve educational practices.

**Cultural and social magic:** As HPV awareness and knowledge may be influenced by religion, culture, and social traditions, examining HPV from the perspectives of awareness, knowledge, and behavior can help identify barriers and facilitators in preventing the virus. Assessing and increasing public awareness of the virus and its consequences through workshops and training campaigns can help diminish taboos, combat neglect of the disease, and change risky behaviors. Additionally, addressing HPV awareness and knowledge can foster discussions and create a social discourse that helps reduce shame and stigma, encourages vaccination, especially among adolescents and young people; and enhances access to and benefits from counseling and health services such as regular tests, screenings, and timely treatment. In short, the cultural and social power of awareness and knowledge about HPV entails creating a supportive, educational, and open environment where people can receive necessary information and take action to maintain their health and that of others. This not only helps reduce the incidence and prevalence of HPV, associated cancers, and genital warts but also improves the quality of life for individuals.

### Advantages and limitations

The first advantage of the present research lies in the combined use of international evidence through the review of evidence, specialists, and target groups in the instrument design and validation process. Second, the analysis of the instrument via various validation methods has enhanced its validity. Third, the questionnaire design encompasses all dimensions associated with a communicable disease (prevalence, risk factors, transmission, symptoms, prevention, screening and diagnosis, consequences, and treatment). Finally, the questionnaire was structured to be self-administered, minimizing interviewer bias.

The study also faced some limitations. The first limitation is related to the sample size. It is recommended that Future studies benefit from larger sample sizes for face validity assessment to enhance statistical power. The second limitation of this study, in line with COSMIN standards, is the absence of a formal Exploratory Factor Analysis (EFA) to confirm the construct validity. This was primarily due to the constraints imposed by the binary response format and the sample size, which was used for a robust known-groups validation. While known-groups validity is a recognized and strong method for providing initial evidence of construct validity by testing hypothesized relationships between groups, it does not replace the need for a full assessment of the underlying factor structure. Therefore, future research must address the factor structure of the HPV-WAK using techniques appropriate for dichotomous data, such as factor analysis of tetrachoric correlations or Item Response Theory (IRT) models, with a larger, independent sample. The third limitation is the possibility of self-reported bias, which was attempted to be minimized by carefully designing the questions, keeping the questionnaires anonymous, and emphasizing data confidentiality. The fourth, like other studies in the field of sexual health, this study has the potential for social desirability bias. However, we minimized this bias by employing three methodological strategies: designing indirect questions (avoiding direct sexual questions), protecting the anonymity of respondents, and asking participants to fill out the questionnaire in a private environment away from other people. Therefore, we believe that this bias in the present study is limited. The generalizability of the findings within the broader Iranian population may be limited. As the study sample was recruited from Dezful University of Medical Sciences, the results may not fully represent the diverse socio-cultural background of women across all regions of Iran. Future studies should employ a multi-regional or national sampling strategy to confirm the instrument’s validity and generalizability throughout the country. Furthermore, cross-cultural validity is the fifth limitation. Our instrument’s cross-cultural validity has not been confirmed, despite its encouraging psychometric qualities in Iran. Therefore, following WHO recommendations for transcultural adaptation, we advise future research to modify and validate this tool in various cultural contexts. This tool was developed taking into account common cultural sensitivities in Islamic countries, but its implementation in other Islamic countries requires a pilot evaluation considering local differences. Therefore, it is suggested that this questionnaire be piloted in neighboring countries that have close cultural similarities with Iran (such as Iraq, Afghanistan, and Pakistan) to assess generalizability. It is recommended that separate validation studies be conducted before using it in other Muslim countries.

## Conclusion

On the basis of the results obtained, the HPV-WAK questionnaire was designed with 29 items. It was created using existing scientific evidence and employs a combined approach of quantitative and qualitative validation methods, achieving good validity and reliability. Since the questionnaire examines all dimensions and stages of the infectious disease HPV, it serves as a valid and efficient instrument for measuring women’s awareness and knowledge in this domain. Therefore, it can be utilized as a foundational instrument in subsequent studies to assess women’s knowledge and awareness of HPV and in intervention studies, including the design of educational programs. Furthermore, health officials can apply it to develop policies and programs that educate and promote community health related to HPV. The questionnaire can also lay the groundwork for further establishing and strengthening collaborations among the fields of medicine, public health, and sociology to address the HPV challenge and women’s educational needs.

## Supporting information

S1 Table Search strategies and selected studies. (DOCX)

S2 Table Content validity results of excluded items. (DOCX)

S3 Table HPV awareness questionnaire. (DOCX)
