# Supplementary material for: Development and validation of the HPV-WAK questionnaire for assessing women’s awareness and knowledge in Iran
Source: PLoS One. 2026 Jan 12;21(1):e0340705. doi: 10.1371/journal.pone.0340705 (PMC12795392; doi:10.1371/journal.pone.0340705)
Supplement: S1 Table — (DOCX) [file pone.0340705.s001.docx]

Table S1. Search strategies

| Pubmed: 148 | |
| --- | --- |
| #1 | (((("Human papillomavirus"[Title/Abstract]) OR ("Human Papillomavirus Viruses"[MeSH Terms])) OR (HPV[Title/Abstract])) OR ("cervical cancer"[Title/Abstract])) OR ("Uterine Cervical Neoplasms"[MeSH Terms]) |
| #2 | (reliability[Title/Abstract]) OR (validity[Title/Abstract]) |
| #3 | ((((((awareness[Title/Abstract]) OR (awareness[MeSH Terms])) OR (knowledge[Title/Abstract])) OR (knowledge[MeSH Terms])) OR (attitude[Title/Abstract])) OR (attitude[MeSH Terms])) OR ("behavioral intention"[Title/Abstract]) |
| #4 | ((((((((Tool[Title/Abstract]) OR (instrument[Title/Abstract])) OR (test[Title/Abstract])) OR (index[Title/Abstract])) OR (checklist[Title/Abstract])) OR (questionnaire[Title/Abstract])) OR (inventory[Title/Abstract])) OR (scale[Title/Abstract])) OR (battery[Title/Abstract]) |
| final | #1 AND #2 AND #3 AND #4 |
| Embase: 239 | |
| #1 | 'human papillomavirus':ta,ab OR hpv:ta,ab OR 'cervical cancer':ta,ab OR 'wart virus'/exp OR 'uterine cervix cancer'/exp |
| #2 | 'reliability':ta,ab OR 'validity':ta,ab OR 'reliability'/exp OR 'validity'/exp |
| #3 | 'awareness':ta,ab OR 'knowledge':ta,ab OR 'attitude':ta,ab OR 'behavioral intention':ta,ab OR 'awareness'/exp OR 'knowledge'/exp OR 'attitude'/exp OR 'behavioral intention'/exp |
| #4 | 'tool':ta,ab OR 'instrument':ta,ab OR 'test':ta,ab OR 'index':ta,ab OR 'checklist':ta,ab OR questionnaire:ta,ab OR 'inventory':ta,ab OR 'scale':ta,ab OR 'battery':ta,ab |
| final | #1 AND #2 AND #3 AND #4 |

Selected studies information:

| **ID** | **First author** | **Country** | **Title** | **Aim of the study** |
| --- | --- | --- | --- | --- |
| 1 | Folahanmi T. Akinsolu | Nigeria | Willingness to Pay for HPV Vaccine among Women Living with HIV in Nigeria | This study aimed to ascertain the variables linked to the inclination to pay for the HPV vaccine among WLWH in Nigeria. This study also sought to assess the knowledge of HPV, cervical cancer, and the HPV vaccine among this population. |
| 2 | Ghadeer K. Al-Shaikh | Saudi Arabia | Knowledge of Saudi female university students regarding cervical cancer and acceptance of the human papilloma virus vaccine | To assess the level of knowledge regarding cervical cancer and the acceptance of the human papilloma virus (HPV) vaccine among Saudi female students in health colleges. |
| 3 | P.A. Anagnostou | Greece | Human papillomavirus knowledge and vaccine acceptability among adolescents in a Greek region | The aim of this research was twofold: (1) develop an instrument to assess knowledge regarding human papillomavirus (HPV) and its vaccine and utilize this instrument to measure knowledge levels of Greek adolescents in Lyceum schools of Western Thessaloniki; and (2) examine the associations of the resultant knowledge measure scores with sociodemographic characteristics. |
| 4 | Usman Ayub Awan | Pakistan | An exploratory study of knowledge, attitudes, and practices toward HPV associated anal cancer among Pakistani population | This study aimed to examine the knowledge, attitudes, and practices (KAP) towards anal cancer screening and HPV of the general population in Pakistan. |
| 5 | David Barrera Ferro | Colombia | Improving intervention design to promote cervical cancer screening among hard-to-reach women: assessing beliefs and predicting individual attendance probabilities in Bogotá, Colombia | The aim is to improve the cost-effectiveness of behavioral interventions aiming to  increase attendance for screening. |
| 6 | Tara F. Bertulfo | United States | An instrument assessing attitudes and beliefs toward human papillomavirus vaccination | The purpose of this study was to test the psychometric properties of a brief attitudes and beliefs toward human papillomavirus (HPV) vaccination instrument in college students. |
| 7 | Feyza Demir Bozkurt | Turkey | Validity and reliability of a Turkish version of the human papillomavirus knowledge scale: a methodological study | The study aimed to test validity and reliability of the Human Papilloma Virus Knowledge Scale (HPV-KS) in Turkish. |
| 8 | O. Cabras | France | Knowledge on human papillomavirus (HPV), HPV screening and HPV vaccine among sexual health clinic patients in Martinique, French West Indies | To assess the level of knowledge about HPV in a population attending a sexual health clinic in the University Hospital of Martinique. |
| 9 | Charitha Gowda | United States | CHIAS: A Standardized Measure of Parental HPV  Immunization Attitudes and Beliefs and Its Associations with Vaccine Uptake | The goals of this study were to determine: (1) whether applying the CHIAS to a national sample of mothers resulted in similar “groupings” of factors (harms, effectiveness, barriers, and uncertainty) as identiﬁed in the original study and (2) whether the associations between the identiﬁed attitudinal constructs and maternal HPV vaccination intention in a nationally representative population were similar to that of the original CHIAS study population. |
| 10 | Hannah Priest Catalano | United States | Using the Theory of Planned Behavior to Predict HPV Vaccination Intentions of College Males | The purpose of this study was to test theory of planned behavior (TPB) constructs in predicting human papillomavirus (HPV) vaccination behavioral intentions of vaccine-eligible college males. |
| 11 | Marlee Grabiel | USA | HPV and HPV Vaccines: The Knowledge Levels, Opinions, and Behavior of Parents | The primary objective of this research was to measure parent knowledge levels and opinions related to the human papillomavirus (HPV) and the two vaccines used to prevent some of its deleterious outcomes and to measure parent behavior in terms of whether or not to have their children vaccinated. Finally, the research intended to explore the antecedents of knowledge, opinions, and behavior. |
| 12 | Annielson de Souza Costa | Brazil | Knowledge gaps and acquisition about HPV and its vaccine among Brazilian medical students | To analyze factors associated with knowledge gaps and acquisition about HPV and its vaccine among medical students. |
| 13 | Jennifer Cunningham-Erves | USA | Development of a Theory-based, Sociocultural Instrument to Assess Black Maternal Intentions to Vaccinate Their Daughters Aged 9 to 12 Against HPV | The purpose of this paper is to describe the development and validation of the Human Papillomavirus Vaccination Survey for Black Mothers with Girls Aged 9 to 12 (HPVS-BM). |
| 14 | Sara Dadipoor | Iran | Predictive power of PEN-3 cultural model in cervical cancer screening among women: a cross-sectional study in South of Iran | The present research aimed to determine the predictors of cervical cancer screening (CCS) based on the PEN-3 model constructs. |
| 15 | E.M. Donadiki | Greece | Health Belief Model applied to non-compliance with HPV vaccine among female university students | This study aimed to investigate the main reasons for refusal of HPV vaccination, and to explore participants’ perceptions and attitudes about HBM constructs (perceived susceptibility, perceived severity, perceived beneﬁts, perceived barriers, cues to action and self-efﬁcacy) among a sample of female university students in Athens. |
| 16 | Ugonma Winnie Dozie | Nigeria | Factors associated with health seeking delay in the screening of cervical cancer among women in Imo state, south Eastern Nigeria | This study aimed to assess the factors associated with health-seeking delay in the screening of cervical cancer among women in Owerri Municipal LGA, Imo State. |
| 17 | Ekhlass M. Eltomy | Egypt | Safe Sex Health Awareness: Knowledge and Attitudes Regarding Male Condom Protection Role against Sexually Transmitted Infections among Minia University Students, Egypt | The aim was to assess knowledge and attitudes regarding male condom protection role against sexually transmitted infections among Minia university students. |
| 18 | A.S. Forster | Australia | Development and validation of measures to evaluate adolescents' knowledge about human papillomavirus (HPV), involvement in HPV vaccine decision-making, self-efﬁcacy to receive the vaccine and fear and anxiety | We describe the development and validation of measures of HPV/HPV vaccination knowledge, fear/anxiety about vaccination, involvement in HPV vaccine decision-making, and self-efﬁcacy with regard to getting the vaccine, designed to evaluate the efﬁcacy of an intervention to affect these domains (collectively termed the HAVIQ: HPV Adolescent Vaccine Intervention Questionnaire). |
| 19 | Tahir Mehmood Khan | Pakistan | Knowledge, attitudes, and perception towards human papillomavirus among university students in Pakistan | This study explores university students' knowledge, attitudes, and perception towards HPV. |
| 20 | Jo Waller | UK, US, and Australia | Validation of a measure of knowledge about human papillomavirus (HPV) using item response theory and classical test theory | While many studies have measured HPV knowledge, none has developed a validated measure for use across countries. We aimed to develop and validate such a measure. |
| 21 | Oyedero L.C | Nigeria | Knowledge of Human Papilloma Virus Self-sampling and Cervical Cancers Screening Uptake among Gynaecology Clinic Attendees in a Tertiary Hospital in Nigeria | this study investigated the level of knowledge Human Papilloma Virus (HPV) Self-sampling and Cervical cancers screening uptake among gynaecology clinic attendees in Obafemi Awolowo University Teaching Hospitals Complex, Ile-Ife, Nigeria. |
| 22 | Gulten Guvenc | Turkey | Health Belief Model Scale for Cervical Cancer and Pap Smear Test: psychometric testing | This study is a report of the development and psychometric testing of the Health Belief Model Scale for Cervical Cancer and the Pap Smear Test. |
| 23 | Gulten Guvenc | Turkey | Health Belief Model Scale for Human Papilloma Virus and its Vaccination: Adaptation and Psychometric Testing | To adapt and psychometrically test the Health Belief Model Scale for Human Papillomavirus and its vaccination (HBMS-HPVV), and to assess human papillomavirus knowledge score (HPV-KS) among female college students. |
| 24 | Saba Haider | Pakistan | Nurse’s knowledge and Awareness Regarding Cervical Cancer and its Prevention in Different Hospitals of Quetta, Pakistan | This study aimed to assess the knowledge and awareness regarding cervical cancer and its prevention among nurses working in different hospitals of Quetta, Pakistan. |
| 25 | Sayward E. Harrison | United States | xamining Associations between Knowledge and Vaccine Uptake Using the Human Papillomavirus Knowledge Questionnaire (HPV-KQ) | Understanding the relationship between human papillomavirus (HPV) knowledge and vaccination behavior is important to inform public health interventions, yet few validated HPV knowledge scales exist. |
| 26 | Maryam Alsadat Hashemipour | Iran | Knowledge of Medical and Dental Iranian Students about the Infection  and Vaccination of Human Papillomavirus | To evaluate the awareness of medical and dental students of Kerman University of Medical Science about the infection and vaccination of the human papillomavirus. |
| 27 | Meera Indracanti | Northwest Ethiopia | Factors Associated with Pre- and Post-Educational Intervention Knowledge Levels of HPV and Cervical Cancer Among the Male and Female University Students, Northwest Ethiopia | This study aimed to understand the factors associated with HPV and cervical cancer knowledge levels of university students before and after an educational intervention. |
| 28 | Sophaphan Intahphuak | Thailand | Factors Influence on Pap Test Screening among Lahu Hill Tribe Women in Remote Area Thailand | This study aimed to determine the factors associated with successful Pap test among Lahu hill tribe women. |
| 29 | Iman A Jaber | Iraq | Knowledge of Women about the Early Detection Methods of Cervical Cancer in | To assess women’s knowledge regarding cervical cancer and early detection methods. |
| 30 | Katarzyna Jaglarz | Poland | Creating and field-testing the questionnaire for the assessment of knowledge about cervical cancer and its prevention among schoolgirls and female students | This study aimed to develop and validate a questionnaire used to assess the level of general knowledge about cervical cancer, its primary and secondary prevention, and to identify sources of information about the disease among schoolgirls and female students. |
| 31 | Phanida Juntasopeepun | Thailand | Development and psychometric evaluation of the Thai Human Papillomavirus Beliefs Scale | developed and evaluated the psychometric properties of the Thai Human Papillomavirus Beliefs Scale. |
| 32 | Seyed Saeed Mazloomy Mahmoodabad | Iran | A questionnaire on factors affecting the precaution adoption process model for cervical cancer – Psychometric properties | This study aims to evaluate the psychometrics of a questionnaire that enquired about the factors involved in the Precaution Adoption Process Model (PAPM) for cervical cancer among suburban women in Bandar Abbas, south of Iran. |
| 33 | André Luciano Manoel | Brasil | Preliminary stages of a cross-cultural Brazilian Portuguese adaptation of a measurement tool for assessing public understanding of human papillomavirus | The objective of this study was to conduct the initial stages of the cross-cultural adaptation to Brazilian Portuguese of a scale to measure the level of knowledge about HPV. |
| 34 | Domenica Matranga | Italy | The vaccination & HPV Knowledge (THinK) questionnaire: a reliability and validity study on a sample of women living in Sicily (southern-Italy) | This study aimed to introduce the Vaccination & HPV Knowledge (THinK) questionnaire to assess knowledge about human papillomavirus (HPV) and  attitude to HPV vaccination. |
| 35 | Annie-Laurie McRee | United States | The Carolina HPV Immunization Attitudes and Beliefs Scale (CHIAS): Scale Development and Associations With Intentions to Vaccinate | developed the Carolina HPV Immunization Attitudes and Beliefs Scale (CHIAS) and explored its factor structure. |
| 36 | Tomasz Milecki | Poland | Polish Medical Students’ Knowledge Regarding Human Papillomavirus’s Ways of Transmission, Risk of Cancer Development, and Vaccination, and Their Intention to Recommend Vaccination | The study aimed to assess the knowledge of medical students regarding Human Papillomavirus’s (HPV) ways of transmission, risk of cancer development, and vaccination against HPV. |
| 37 | S. Mojahed | Iran | Attitude and knowledge of Iranian female nurses about Human Papillomavirus infection and cervical cancer: a cross-sectional survey | This study aimed to evaluate awareness and knowledge of HPV infection and vaccines and to assess the attitude and approach toward these vaccines among female nurses at Shahid Sadoughi University of Medical Sciences, Yazd, Iran. |
| 38 | J. Moodley | South Africa | Development and validation of the African Women Awareness of CANcer (AWACAN) tool for breast and cervical cancer | This study aimed to develop and validate the African Women Awareness of CANcer (AWACAN) tool to measure awareness of breast and cervical cancer in Sub-Saharan Africa (SSA). |
| 39 | Connie K. Y. Nguyen-Truong | Vietnam | Adaptation and Testing of Instruments to Measure Cervical Cancer Screening Factors Among Vietnamese Immigrant Women | We sought to (a) develop a culturally sensitive Vietnamese translation of the Revised Susceptibility, Benefits, and Barriers Scale; Cultural Barriers to Screening Inventory; Confidentiality Issues Scale; and Quality of Care from the Health Care System Scale and (b) examine the psychometric properties. |
| 40 | Chukwunonyerem C. Ogwunga | Nigeria | Knowledge and Attitude of Female Students of Tertiary Institutions in Imo State, Nigeria Towards Cervical Cancer and Its Screening | This study was carried out to ascertain the knowledge and attitude of female students in four tertiary institutions in Imo State towards cervical cancer and its screening. |
| 41 | E. Ozdemir | Turkey | Validation of the Turkish Cervical Cancer and Human Papilloma Virus Awareness Questionnaire | The aim of this study was to determine the validity and reliability of the ‘Cervical Cancer and Human Papilloma Virus Awareness Questionnaire’ among fertility age women by adapting the scale into Turkish. |
| 42 | Samara Perez | Canada | Development and Validation of the Human Papillomavirus Attitudes and Beliefs Scale in a National Canadian Sample | The study aimed to develop a comprehensive, validated and reliable HPV vaccination attitudes and beliefs scale among parents of boys. |
| 43 | Serena Donati | Italy | Knowledge, Attitude and practice in primary and secondary cervical cancer prevention among young adult Italian women | The survey aimed to evaluate knowledge, attitudes, practices (KAP) regarding cervical cancer infection, Pap testing, HPV, and HPV Vaccination among women aged 18–26 years. |
| 44 | Lilliam M. Pinzon | US  Mexico | Cross-cultural adaptation of a Spanish version of a previously validated HPV survey that evaluates dental students’ knowledge, perception and clinical practices in Latin America | The present study adapted and performed pretesting of a multi-scale survey evaluating knowledge, perceptions, and clinical practices regarding HPV and HPV-OPC for Latin American Spanish-speaking populations. |
| 45 | Hannah M. Priest | USA | Social Cognitive Theory Predictors of Human Papillomavirus Vaccination Intentions of College Men at a Southeastern University | The purpose of this study was to use social cognitive theory to predict human papillomavirus (HPV) vaccination intentions of college men attending a large, southeastern university. |
| 46 | Rosethe Rimande-Joel | Nigeria | Knowledge, Belief and Practice of Cervical Cancer Screening and Prevention among Women of Taraba, North-East Nigeria | The study aimed to establish the cervical cancer knowledge, belief and prevention/screening practices among women in Taraba, North-East Nigeria. |
| 47 | Rodziah Romli | Malaysia | Translation, Cross-Cultural Adaptation, and Validation of the Malay Version of the Protection Motivation Theory Scale Questionnaire for Pap Smear Screening | This study was conducted to validate an adapted instrument based on the protection motivation theory (PMT) in the Malay language, aiming to evaluate the motivational factors for Pap smear screening among women. |
| 48 | Sneha Sethi | Australia | Development and validation of an HPV infection knowledge assessment scale among Aboriginal and Torres Strait Islander Peoples | to examine the psychometric properties of the HPV Knowledge Tool (HPV-KT |
| 49 | María Teresa Urrutia | Chile | Development and testing of the questionnaire CEC-61: Knowledge about cervical cancer in Chilean adolescents | This study aimed to develop and test a new questionnaire concerning knowledge of cervical cancer. |
| 50 | Mary E. Vanc | New Mexico State | Development and Validation of the Cervical Cancer Knowledge and Beliefs of Appalachian Women Questionnaire | This study aimed to develop, test, and use a new instrument that quantifies the cervical cancer knowledge, behavior, and beliefs of Appalachian women. |
| 51 | Jo Waller | USA, Australia, and UK | Validation of a measure of knowledge about human papillomavirus (HPV) using item response theory and classical test theory | This study aimed to develop a valid and reliable measure of knowledge about HPV, HPV testing and HPV vaccination suitable for use across different countries |
| 52 | Karen Patricia Williams | USA | Bringing the Real World to Psychometric Evaluation of Cervical Cancer Literacy Assessments with Black, Latina, and Arab Women in Real-World Settings | Validating the instrument with Black, Latina, and Arab women in English, Spanish, and Arabic provides baseline measures to assist in the development and refinement of cancer education and interventions in three distinct racial/ethnic groups of women. |
| 53 | Tingting Xin | China | Using Delphi method to develop Chinese women’s cervical cancer screening intention scale based on planned behavior theory | The Delphi method to develop a scientifically sound and clinically useful Chinese cervical cancer |
| 54 | Soudabeh Yarmohammadi | Iran | Designing and psychometric assessment of the scale of factors influencing HPV vaccine uptake behaviors in young adults | psychometric assessment of the scale of factors influencing HPV vaccine uptake behaviors |
| 55 | Ephrem Yohannes | Ethiopia | Knowledge and attitude toward human papillomavirus vaccination and associated factors among adolescent school girls in Ambo town, Ethiopia, 2021: A multicenter cross‐sectional study | to assess knowledge and attitudes regarding HPV vaccination and related factors |
| 56 | Lingmin Zhang | China | Sociocultural–psychological predictors inﬂuencing parents’ decision-making regarding HPV vaccination for their adolescent daughters in mainland China: An extended TPB model | to examine the influence of sociocultural-psychological predictors, including exposure to HPV-related stories (positive/negative), affective reactions (pride/regret), injunctive norms on the Internet and perceived moral obligation, on parents’ HPV vaccination-related decision-making for girls aged 13–15 year |
