# Supplementary material for: Development and validation of the HPV-WAK questionnaire for assessing women’s awareness and knowledge in Iran
Source: PLoS One. 2026 Jan 12;21(1):e0340705. doi: 10.1371/journal.pone.0340705 (PMC12795392; doi:10.1371/journal.pone.0340705)
Supplement: S2 Table — (DOCX) [file pone.0340705.s002.docx]

**Table S2. Content validity results of excluded items**

|  | Item | CVR | Evaluation |
| --- | --- | --- | --- |
| 1 | Most sexually active people will get HPV at some point in their lives. | 0.6 | Reject |
| 2 | There are several types of HPV. | 0.6 | Reject |
| 3 | Sometimes, HPV infection can last for years. | 0.6 | Reject |
| 4 | Smoking is a risk factor for HPV infection. | 0.2 | Reject |
| 5 | Smoking increases the risk of cervical cancer if a woman is infected with HPV. | 0.6 | Reject |
| 6 | Only men can transmit HPV. | 0.6 | Reject |
| 7 | Genital warts are the main symptom of HPV infection. | 0.6 | Reject |
| 8 | Vaginal bleeding during sex can be a sign of cervical cancer. | 0.4 | Reject |
| 9 | Blood in the urine or stool is a sign of cervical cancer. | 0.6 | Reject |
| 10 | HPV vaccines are most effective when given to people who have never had sex. | 0.6 | Reject |
| 11 | The HPV vaccine has no side effects. | 0.6 | Reject |
| 12 | After receiving a full dose of the HPV vaccine, sexual intercourse with multiple partners is allowed. | 0.6 | Reject |
| 13 | HPV vaccination is only needed for adult women (over 30 years of age). | 0.6 | Reject |
| 14 | The HPV vaccine requires three doses. | 0.6 | Reject |
| 15 | HPV screening lets us know if the HPV vaccine is needed. | 0.6 | Reject |
| 16 | The HPV test can tell you how long you have had an HPV infection. | 0.6 | Reject |
| 17 | Genital warts affect both men and women. | 0.6 | Reject |
| 18 | Genital warts tend to occur in people over the age of 35. | 0.6 | Reject |
| 19 | HPV can cause cancer in areas such as the head and neck. | 0.6 | Reject |
| 20 | HPV can be treated with antibiotics. | 0.4 | Reject |
| 21 | HPV usually does not require any treatment. | 0.6 | Reject |
