# Supplementary material for: Development and validation of the HPV-WAK questionnaire for assessing women’s awareness and knowledge in Iran
Source: PLoS One. 2026 Jan 12;21(1):e0340705. doi: 10.1371/journal.pone.0340705 (PMC12795392; doi:10.1371/journal.pone.0340705)
Supplement: S3 Table — (DOCX) [file pone.0340705.s003.docx]

**Table S3. Final HPV-WAK questionnaire (English version)**

|  | **Item** | **correct** | **wrong** | **I do not know** |
| --- | --- | --- | --- | --- |
| 1 | Human papillomavirus infection is very rare. |  |  |  |
| 2 | Men are not at risk of getting HPV. |  |  |  |
| 3 | Early initiation of sexual activity reduces the risk of getting HPV. |  |  |  |
| 4 | The risk of obtaining HPV is increased by having several sexual partners. |  |  |  |
| 5 | Being in a monogamous relationship eliminates the risk of getting HPV. |  |  |  |
| 6 | Sexual interaction can transmit HPV. |  |  |  |
| 7 | The risk of getting HPV is reduced by using a condom. |  |  |  |
| 8 | HPV can only be transmitted from a carrier who has obvious symptoms. |  |  |  |
| 9 | During pregnancy and childbirth, HPV can be passed from mother to child. |  |  |  |
| 10 | Skin contact in the genital area can spread HPV. |  |  |  |
| 11 | The majority of HPV-positive individuals are unaware of their condition because it does not show obvious signs or symptoms. |  |  |  |
| 12 | Abnormal bleeding between periods is a symptom of cervical cancer. |  |  |  |
| 13 | Only sexually active women should get the HPV vaccine. |  |  |  |
| 14 | Every sexually transmitted infection can be prevented with the HPV vaccine. |  |  |  |
| 15 | One way to prevent cervical cancer is to get the HPV vaccine. |  |  |  |
| 16 | If a person has been vaccinated, they will never get HPV. |  |  |  |
| 17 | A person who has been infected with HPV should still get vaccinated. |  |  |  |
| 18 | Vaccination prevents certain types of HPV. |  |  |  |
| 19 | Good sexual hygiene alone can prevent cervical cancer. |  |  |  |
| 20 | Women who have received the HPV vaccine do not need to have routine screening exams. |  |  |  |
| 21 | The HPV test and Pap smear can be conducted together. |  |  |  |
| 22 | A woman will undoubtedly get cervical cancer if her HPV test is positive. |  |  |  |
| 23 | Cervical cancer is curable if caught early. |  |  |  |
| 24 | The only way to diagnose cervical cancer is with a Pap smear. |  |  |  |
| 25 | HPV can cause genital warts. |  |  |  |
| 26 | HPV only causes cervical cancer in women. |  |  |  |
| 27 | HPV always causes cancer. |  |  |  |
| 28 | There is no specific treatment for HPV. |  |  |  |
| 29 | The only cure for cervical cancer is to remove the uterus. |  |  |  |
